# 超高效液相色谱-串联质谱法测定尿液中血栓素A_2_的3种代谢物和8-异前列腺素F_2α_

**DOI:** 10.3724/SP.J.1123.2024.02004

**Published:** 2025-02-08

**Authors:** Sijia LIU, Furong ZHAO, Yalian ZHANG, Xiaoyu SUN, Mengmeng ZHANG, Kun HOU, Yunfeng CAO

**Affiliations:** 1.锦州医科大学药学院,辽宁锦州121001; 1. School of Pharmacy, Jinzhou Medical University, Jinzhou 121001, China; 2.上海市生物医药技术研究院,上海市疾病与健康基因组学重点实验室,国家卫生健康委员会计划生育药具重点实验室,上海200237; 2. Shanghai-MOST Key Laboratory of Health and Disease Genomics, NHC Key Lab of Reproduction Regulation, Shanghai Institute for Biomedical and Pharmaceutical Technologies, Shanghai 200237, China; 3.锦州医科大学,辽宁省肿瘤临床代谢组学重点实验室,辽宁锦州121001; 3. Liaoning Provincial Key Laboratory of Clinical Oncology Metabonomics, Jinzhou Medical University, Jinzhou 121001, China; 4.大连博源医学科技有限公司,辽宁大连116011; 4. Dalian Boyuan Medical Technology Co. Ltd., Dalian 116011, China

**Keywords:** 超高效液相色谱-串联质谱, 2,3-地诺血栓素B_2_, 11-脱氢-2,3-地诺血栓素B_2_, 11-脱氢血栓素B_2_, 8-异前列腺素F_2α_, 尿液, 缺血性脑卒中, ultra performance liquid chromatography-tandem mass spectrometry (UPLC-MS/MS), 2,3-dinor thromboxane B_2_(2,3-dinor-TXB_2_), 11-dehydro-2,3-dinor thromboxane B_2_(11-dh-2,3-dinor-TXB_2_), 11-dehydro thromboxane B_2_(11-dh-TXB_2_), 8-iso-prostaglandin F_2α_(8-iso-PGF_2α_), urine, ischemic stroke

## Abstract

建立了同时测定人体尿液中血栓素A_2_的3种代谢物(2,3-地诺血栓素B_2_、11-脱氢-2,3-地诺血栓素B_2_和11-脱氢血栓素B_2_)和8-异前列腺素F_2α_的超高效液相色谱-串联质谱检测方法。尿液经盐酸酸化至pH 2.0~4.0,经C18固相萃取柱净化后测定。使用ACQUITY UPLC^® ^ BEH Phenyl色谱柱(50 mm×2.1 mm, 1.7 μm),以含0.002%氨水的2 mmol/L乙酸铵水溶液和乙腈作为流动相进行梯度洗脱,流速为0.3 mL/min,柱温为40 ℃,采用电喷雾离子源,在负离子和多反应监测模式下分析4 min,内标法定量。4种目标化合物在各自范围内线性关系良好,相关系数(*R*^2^)大于0.99;方法检出限为2,3-地诺血栓素B_2_ 0.02 ng/mL,其余目标化合物0.01 ng/mL;定量限为2,3-地诺血栓素B_2_ 0.1 ng/mL,其余目标化合物0.05 ng/mL;实际尿液中,4种目标物在定量限水平下的加标回收率为91.48%~104.87%,低、中、高水平下的加标回收率为92.95%~104.90%,日内精密度为2.79%~13.01%,日间精密度为4.45%~13.67%。应用该方法测定健康人群和缺血性脑卒中患者血栓素A_2_的3种代谢物和8-异前列腺素F_2α_的含量,并用肌酐进行校正。对目标化合物进行二元Logistic回归分析并绘制受试者工作特征曲线(ROC曲线)用于诊断,血栓素A_2_(血栓素A_2_的3种代谢物加和)的曲线下面积(AUC)为0.849, 8-异前列腺素F_2α_的AUC为0.775,具有较好的临床价值,有望辅助缺血性脑卒中的早期筛查诊断。

## Abstract (en)

Thromboxane A_2_ (TXA_2_), a prothrombotic factor that induces platelet aggregation and thrombosis, acts as a vasoconstrictor by activating TXA_2_ receptors (TP receptors). TXA_2_ is extremely unstable and metabolizes into three major metabolites: 2,3-dinor thromboxane B_2_ (2,3-dinor-TXB_2_), 11-dehydro TXB_2_(11-dh-TXB_2_), and 11-dehydro-2,3-dinor TXB_2_(11-dh-2,3-dinor-TXB_2_). 8-Iso-prostaglandin F_2α_(8-iso-PGF_2α_), a prostaglandin-like compound widely considered the best biomarker of oxidative stress, can also activate TP receptors. The accurate quantification of TXA_2_ metabolites and 8-iso-PGF_2α_ is critical in cardiovascular disease (CVD).

In this study, a method based on ultra performance liquid chromatography-tandem mass spectrometry (UPLC-MS/MS) was developed for the simultaneous determination of 2,3-dinor-TXB_2_,11-dh-2,3-dinor-TXB_2_, 11-dh-TXB_2_, and 8-iso-PGF_2α_ in human urine. Urine samples were collected from healthy volunteers and patients with CT- or MRI-confirmed ischemic stroke occurring less than 48 h earlier, and cryopreserved at -80 ℃ within 1 h after collection. The urine samples were thawed at room temperature and acidified to pH 2.0-4.0 using hydrochloric acid. The supernatant was collected after centrifugation. A total of 1 mL of each urine sample was added with 100 μL of the internal standard working solution and mixed well. The samples were loaded onto a C18 SPE column (50 mg). The SPE cartridges were preconditioned with 500 μL of methanol and then equilibrated with 500 μL of water. After sample loading, the SPE cartridges were washed with 500 μL of water, 500 μL of 5% methanol aqueous solution containing 0.5% (v/v) ammonia, and 500 μL of 5% methanol aqueous solution containing 2% (v/v) formic acid. The cartridges were dried, and the analytes were eluted with 400 μL of methanol. The eluents were dried and subsequently reconstituted with 50 μL of 13% acetonitrile aqueous solution. After filtration through a filter membrane, the samples were analyzed on an ACQUITY UPLC^®^ BEH phenyl column (50 mm×2.1 mm, 1.7 μm) via gradient elution using 2 mmol/L ammonium acetate aqueous solution containing 0.002% (v/v) ammonia and acetonitrile as the mobile phases. The flow rate was 0.3 mL/min, and the column temperature was 40 ℃. The analytes were determined in negative electrospray ionization and multiple-reaction monitoring modes. The four target compounds showed satisfactory linearity within the relevant ranges, with linear correlation coefficients (*R*^2^) greater than 0.99. The limits of detection of the method were 0.02 ng/mL for 2,3-dinor-TXB_2_ and 0.01 ng/mL for 11-dh-2,3-dinor-TXB_2_, 11-dh-TXB_2_, and 8-iso-PGF_2α_. The limits of quantification were 0.1 ng/mL for 2,3-dinor-TXB_2_ and 0.05 ng/mL for 11-dh-2,3-dinor-TXB_2_, 11-dh-TXB_2_, and 8-iso-PGF_2α_. In actual urine, the recovery rates at the LOQ level were in the range of 91.48%-104.87%. The recovery rates at low, medium, and high levels were in the range of 92.95%-104.90%. The intra- and inter-day precisions were in the range of 2.79%-13.01% and 4.45%-13.67%, respectively. The relative error (RE) between the average peak area of the mixed matrix and the sum of the ratios of the pure solution and urine matrices was within ±20%. The samples were stable at 4 ℃ for 24 h and at -70 ℃ for 10 d. The developed method is the first to realize the simultaneous determination of 2,3-dinor-TXB_2_, 11-dh-2,3-dinor-TXB_2_, 11-dh-TXB_2_, and 8-iso-PGF_2α_ in urine. The method was used to determine the concentrations of 2,3-dinor-TXB_2_, 11-dh-2,3-dinor-TXB_2_, 11-dh-TXB_2_, and 8-iso-PGF_2α_ in healthy controls and patients with ischemic stroke, and the results were corrected using creatinine. Binary logistic regression analysis was used to construct the prediction model, and a receiver operating characteristic (ROC) curve was drawn to evaluate the clinical diagnostic ability of the method for the target compounds. TXA_2_ was calculated as the sum of its three metabolites. The area under the curve (AUC) of TXA_2_ was 0.849, and the method sensitivity and specificity were 69.2% and 92.3%, respectively. The AUC of 8-iso-PGF_2α_ was 0.775, and the method sensitivity and specificity were 84.6% and 76.9%, respectively. The proposed method has good clinical value and is expected to assist in the early screening and diagnosis of ischemic stroke.

血栓素A_2_(TXA_2_)是花生四烯酸经酶催化的代谢物^[1]^,是一种促血栓形成因子,可诱导血小板聚集和血栓形成,也是一种血管收缩因子,通过激活血栓素A_2_受体(TP受体)发挥作用^[2]^。然而TXA_2_非常不稳定,会迅速生成生物活性不强的血栓素B_2_(TXB_2_)。虽然TXB_2_相对稳定且较容易在血浆和尿液中检测,但其还会进一步代谢生成更稳定的代谢物^[3]^。TXB_2_的稳定代谢物常作为生物标志物来评估体内TXA_2_的形成,主要有脱氢生成的11-脱氢血栓素B_2_(11-dh-TXB_2_)和β-氧化生成的2,3-地诺血栓素B_2_(2,3-dinor-TXB_2_)^[3,4]^。另有研究发现β-氧化和脱氢均生成的代谢物11-脱氢-2,3-地诺血栓素B_2_(11-dh-2,3-dinor-TXB_2_)^[5]^的检测也非常重要,其可能在动脉粥样硬化血栓形成中起关键作用^[6]^,因此本研究将11-dh-2,3-dinor-TXB_2_纳入检测。

8-异前列腺素F_2α_(8-iso-PGF_2α_)是一种类似前列腺素的化合物,主要来源于花生四烯酸的脂质过氧化^[7]^,是氧化应激的最佳生物标志物^[8]^,同样也可激活TP受体发挥作用^[9]^。

已有研究发现TXA_2_和8-iso-PGF_2α_参与动脉粥样硬化的发生和发展^[10]^,且均为评估缺血再灌注损伤的潜在生物学指标^[11]^。8-iso-PGF_2α_可导致阿司匹林耐药^[9]^, TXA_2_的稳定代谢物可用于评估阿司匹林的抗血小板作用以确保抗血栓疗效^[12,13]^。在中国,因心脑血管疾病(CVD)而死亡的人数从2005年的309万人增加到2020年的458万人,其中缺血性心脏病(IHD)、出血性卒中(HS)和缺血性卒中(IS)是CVD死亡的前三位原因^[14]^。因此,针对CVD,尿液中TXA_2_代谢物和8-iso-PGF_2α_的准确定量至关重要。

在采集血样的过程中,因血管损伤,TXA_2_的含量会急剧升高^[15]^。在体外条件下,血浆中的脂质会继续发生自动氧化形成异前列腺素^[16]^。因此对于8-iso-PGF_2α_和TXA_2_的代谢物11-dh-TXB_2_、2,3-dinor-TXB_2_、11-dh-2,3-dinor-TXB_2_的检测,尿液相较于血浆更具实际意义。

目前对8-iso-PGF_2α_和TXA_2_的代谢产物11-dh-TXB_2_、2,3-dinor-TXB_2_、11-dh-2,3-dinor-TXB_2_可采用的检测方法有酶免疫分析法(EIA)、液相色谱法(LC)、气相色谱-质谱法(GC-MS)和液相色谱-质谱法(LC-MS)。其中EIA的专属性较差,易发生交叉反应,出现假阳性的结果,且不适用于多个分析物。例如单克隆抗体酶联免疫吸附测定中,11-dh-2,3-dinor-TXB_2_影响尿中11-dh-TXB_2_测定的准确性和临床实用性^[17]^。LC和GC-MS需衍生化,过程繁琐^[18,19]^。本研究采用具有较好灵敏度和专属性的超高效液相色谱-串联质谱法(UPLC-MS/MS),同时测定尿液中11-dh-TXB_2_、2,3-dinor-TXB_2_、11-dh-2,3-dinor-TXB_2_和8-iso-PGF_2α_。

## 1 实验部分

### 1.1 仪器、试剂与材料

超高效液相色谱仪(配有LC-40D X3液相色谱泵、DGU-403和DGU-405脱气机、CTO-40C柱温箱和SIL-40C X3自动进样器)、8060NX三重四极杆质谱仪(Shimadzu,日本);高速台式冷冻离心机(湘仪,中国);氮气吹扫仪(杭州奥盛,中国);万分之一天平(Precisa,瑞士)。

8-异前列腺素F_2α_(纯度≥98%)、2,3-地诺血栓素B_2_(纯度≥95%)、11-脱氢-2,3-地诺血栓素B_2_(纯度≥98%)、11-脱氢血栓素B_2_(纯度≥98%)、8-异前列腺素F_2α_-d4(纯度≥99%)、2,3-地诺血栓素B_2_-d9(纯度≥99%)、11-脱氢血栓素B_2_-d4(纯度≥99%)(Cayman,美国)。

盐酸(科密欧,中国);乙酸铵、甲醇、乙腈、0.45 μm滤膜(Merck,德国);氨水(阿拉丁,中国); 10×0.01 mol/L PBS缓冲液(索莱宝,中国); C18固相萃取柱(50 mg)(月旭,中国); 96孔板(耐思,中国)。

### 1.2 标准溶液配制

用乙腈将各标准品和内标稀释成质量浓度为1 μg/mL的标准储备液和内标储备液,于-70 ℃保存备用。分别移取适量各标准储备液和内标储备液,用乙腈稀释成混合标准储备液和混合内标储备液,现用现配。

用乙腈稀释混合标准储备液和混合内标储备液得到系列标准工作液和内标混合标准工作液,8-iso-PGF_2α_、11-dh-2,3-dinor-TXB_2_、11-dh-TXB_2_质量浓度为0.5、1.0、2.0、4.0、8.0、20、40、75、100 ng/mL, 2,3-dinor-TXB_2_质量浓度为1.0、2.0、4.0、8.0、16、40、80、150、200 ng/mL, 8-iso-PGF_2α_-d4和2,3-dinor-TXB_2_-d9质量浓度为20 ng/mL, 11-dh-TXB_2_-d4质量浓度为10 ng/mL。

### 1.3 实验条件与方法

#### 1.3.1 样本收集

研究对象为锦州医科大学附属第一医院13例缺血性脑卒中首次发病患者和26例健康体检者(对照组)。缺血性脑卒中患者通过CT或MRI确认脑卒中病灶,首次发病时间小于48 h。所有患者发病后48 h内均符合《中国急性缺血性脑卒中诊治指南2018》^[20]^:(1)急性起病;(2)局灶神经功能缺损(一侧面部或肢体无力或麻木,语言障碍等),少数为全面神经功能缺损;(3)影像学出现责任病灶或症状/体征持续24 h以上;(4)排除非血管性病因;(5)脑CT/MRI排除脑出血。两组年龄、性别和体重指数(BMI)无统计学差异(*P*>0.05)。所有样品采集后1 h内于-80 ℃冷冻保存。研究所涉及尿液样本均经锦州医科大学附属第一医院伦理委员会批准(KYLL2023157)。

#### 1.3.2 样品前处理

冷冻尿液在室温解冻后加入盐酸调节pH 2.0~4.0, 3000 r/min离心5 min,取上清1 mL加入100 μL内标混合标准工作液,2000 r/min涡旋5 min后上样至依次用500 μL甲醇和500 μL水活化平衡的固相萃取小柱。分别用500 μL水、含0.5%氨水的5%甲醇水溶液、含2%甲酸的5%甲醇水溶液依次淋洗,用400 μL甲醇洗脱。洗脱液在40 ℃下经氮气吹干,50 μL 13%乙腈水溶液复溶,混匀后过0.45 μm滤膜后进样。

#### 1.3.3 色谱条件

色谱柱:ACQUITY UPLC^® ^ BEH Phenyl色谱柱(50 mm×2.1 mm, 1.7 μm,美国Waters公司);柱温:40 ℃;样品室温度:4 ℃;流动相:A为含0.002%氨水的2 mmol/L乙酸铵水溶液,B为乙腈;流速:0.3 mL/min。梯度洗脱程序:0~0.5 min, 13%B~14%B; 0.5~1.5 min, 14%B~28%B; 1.5~2.1 min, 28%B~30%B; 2.1~2.5 min, 30%B~35%B; 2.5~2.7 min, 35%B~36%B; 2.7~2.8 min, 36%B~40%B; 2.8~2.81 min, 40%B~95%B; 2.81~3.0 min, 95%B; 3.0~3.1 min, 95%B~13%B; 3.1~4.0 min, 13%B。进样量:5 μL。

#### 1.3.4 质谱条件

采用电喷雾电离方式进行离子化,负离子、多反应监测模式扫描。扫描时间:4 min;雾化气流量:3 L/min;加热气流量:10 L/min;接口温度:400 ℃;脱溶剂管温度:200 ℃;加热块温度:400 ℃;干燥气流量:10 L/min。其他质谱参数见表1。

**表1:** 目标化合物和内标的质谱参数

Compound	Retention time/min	Precursor ion (*m/z*)	Product ion (*m/z*)	Q1 Pre Bias/V	CE/ V	Q3 Pre Bias/V	IS
2,3-Dinor thromboxane B_2_	2.0	341.35	167.10^*^	10.0	12.0	14.0	2,3-dinor-TXB_2_-d9
(2,3-dinor-TXB_2_)			123.05	10.0	19.0	21.0	
11-Dehydro-2,3-dinor thromboxane B_2_	2.2	339.30	277.35^*^	19.0	11.0	27.0	11-dh-TXB_2_-d4
(11-dh-2,3-dinor-TXB_2_)			235.30	10.0	14.0	14.0	
8-Iso prostaglandin F_2α_	2.3	353.35	193.20^*^	18.0	26.0	11.0	8-iso-PGF_2α_-d4
(8-iso-PGF_2α_)			309.15	11.0	20.0	13.0	
11-Dehydro thromboxane B_2_	2.5	367.30	304.95	18.0	16.0	19.0	11-dh-TXB_2_-d4
(11-dh-TXB_2_)			161.10^*^	10.0	20.0	15.0	
2,3-dinor-TXB_2_-d9	2.0	350.35	167.20^*^	17.0	12.0	10.0	
			140.90	18.0	15.0	21.0	
8-iso-PGF_2α_-d4	2.3	357.35	313.15	18.0	21.0	20.0	
			197.30^*^	18.0	26.0	20.0	
11-dh-TXB_2_-d4	2.5	371.35	165.40^*^	20.0	20.0	10.0	
			309.10	22.0	18.0	19.0	

* Quantitative ion.

#### 1.3.5 统计学分析

运用二元Logistic回归分析构建预测模型,绘制受试者工作特征曲线(ROC曲线)评价目标化合物的临床诊断能力,数据分析采用SPSS25.0软件。

## 2 结果与讨论

### 2.1 质谱及色谱条件优化

目标化合物为弱酸性物质,结构中含有羟基及羧基基团,易失氢产生相对分子质量为M-1的母离子,因此采用负离子模式。目标化合物和内标的标准溶液经前体离子搜索、前体*m/z*调整、产物*m/z*选择、CE优化和详细优化、产物*m/z*调整、Q1 prerod偏差优化和Q3 prerod偏差优化后得到优化报告。最终优化参数见表1。

以乙腈为有机相,对比了水相和添加不同浓度甲酸铵、乙酸铵时的峰形及响应情况。结果显示,直接用水作为流动相,11-dh-TXB_2_响应很高,但其他3种化合物响应低;添加甲酸铵,各目标化合物色谱峰右侧出现肩峰;添加浓度为0.5、1、2 mmol/L的乙酸铵,随乙酸铵浓度增大,8-iso-PGF_2α_响应降低,但拖尾减轻且化合物色谱峰变窄。由于目标化合物在负离子模式下检测,为保证体系的稳定,因此在水相中加入0.002%的氨水。最终确定流动相水相为含0.002%氨水的2 mmol/L乙酸铵水溶液。4种目标化合物的提取离子色谱图见图1。

**图 1:**
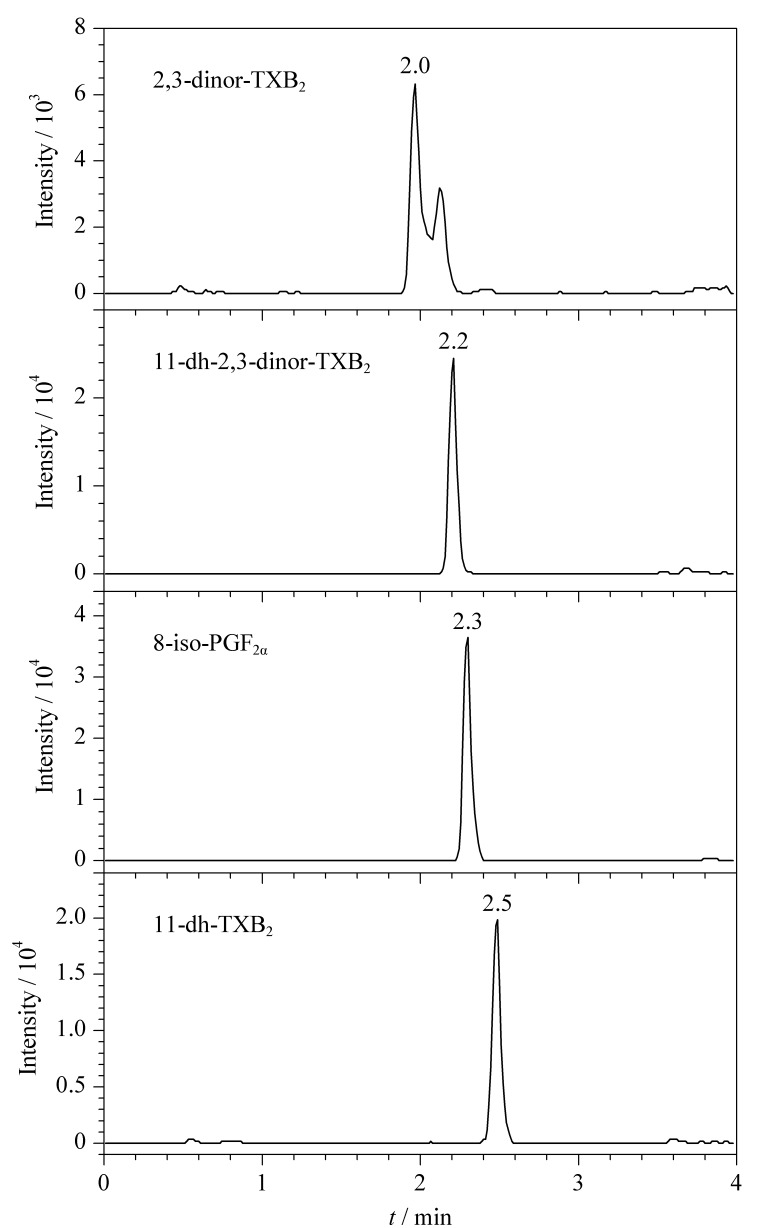
4种目标化合物混合标准溶液(5 ng/mL)的提取离子色谱图

### 2.2 方法学考察

待测化合物属于内源性物质,本研究依据《中国药典》(2020版)中“生物样品定量分析方法验证指导原则”,采用pH为2.0~4.0的0.01 mol/L PBS作为空白基质,向其中添加标准品进行方法学验证,考察了方法的线性范围、基质效应、回收率、精密度和稳定性。

#### 2.2.1 线性范围、检出限与定量限

根据各目标分析物峰面积和内标峰面积的比值*y*对质量浓度*x*(ng/mL)进行权重线性回归,拟合工作曲线,权重为1/*x*^2^,线性方程见表2。4种目标化合物在相应线性范围内线性关系良好,相关系数(*R*^2^)均大于0.99。检出限(LOD)以信噪比(*S/N*)≥3确定,定量限(LOQ)以*S/N*≥10确定,2,3-dinor-TXB_2_检出限为0.02 ng/mL,定量限为0.1 ng/mL,其余目标化合物检出限为0.01 ng/mL,定量限为0.05 ng/mL。

**表2:** 4种目标化合物的线性方程、相关系数、线性范围、检出限和定量限

Compound	Linear equation	*R*^2^	Linear range/(ng/mL)	LOD/(ng/mL)	LOQ/(ng/mL)
8-iso-PGF_2α_	*y*=1.22740*x*-0.00167152	0.9993	0.05-10	0.01	0.05
2,3-dinor-TXB_2_	*y*=0.216489*x*+0.00263317	0.9986	0.10-20	0.02	0.10
11-dh-2,3-dinor-TXB_2_	*y*=1.15989*x*+0.00819179	0.9987	0.05-10	0.01	0.05
11-dh-TXB_2_	*y*=1.93201*x*+0.0226548	0.9996	0.05-10	0.01	0.05

*y*: chromatographic peak area ratio of each target compound to corresponding internal standard; *x*: mass concentration of target compound, ng/mL.

#### 2.2.2 基质效应评价

基质效应参照Cao等^[21]^的方法,即选取目标分析物纯溶液样本、尿液样本以及二者混合液样本进行基质效应评价。选取中水平(2,3-dinor-TXB_2_, 1.80 ng/mL;其余3种化合物,0.90 ng/mL)的目标分析物纯溶液样本(平行做6份),计算目标物和内标响应比值的均值*y_s_*;选取6份尿液样本(采集自不同志愿者),分别计算目标物和内标响应的比值*y_x_*;混合液样本为上述两种基质的3∶7或7∶3(v/v)混合物,分别计算目标物和内标响应的比值*y*_3∶7_或*y*_7∶3_。偏差(RE)_3∶7_=1-*y*_3∶7_/(*y_s_*×0.3+*y_x_*×0.7),偏差_7∶3_=1-*y*_7∶3_/(*y_s_*×0.7+*y_x_*×0.3)。结果表明,以上RE均在±20%以内,说明基质效应并不影响目标化合物的准确定量。

#### 2.2.3 回收率和精密度

以实际尿样进行4种化合物定量限、低、中、高4个水平的加标试验,每个水平进行6次平行试验,计算回收率和日内精密度;在不同天进行上述实验,计算日间精密度。如表3所示,定量限水平下加标回收率为91.48%~104.87%,低、中、高水平加标回收率为92.95%~104.90%,日内精密度为2.79%~13.01%,日间精密度为4.45%~13.67%。

**表3:** 4种目标化合物在4个水平下的加标回收率、日内和日间精密度(*n*=6)

Compound	Spiked/ (ng/mL)	Average recovery /%	RSDs/%	
			Intra-day	Inter-day
8-iso-PGF_2α_	0.05	104.53	7.18	8.06
	0.10	98.43	7.90	6.97
	0.90	92.95	5.62	5.90
	8.00	97.33	4.36	4.78
2,3-dinor-TXB_2_	0.10	101.47	4.83	9.67
	0.20	100.93	6.64	8.88
	1.80	104.73	3.89	6.78
	16.00	101.13	2.79	4.45
11-dh-2,3-dinor-TXB_2_	0.05	91.48	10.97	11.72
	0.10	97.60	3.37	9.61
	0.90	100.57	8.13	7.74
	8.00	95.50	5.86	6.37
11-dh-TXB_2_	0.05	104.87	13.01	12.31
	0.10	104.90	3.42	13.67
	0.90	98.85	8.26	7.68
	8.00	96.53	4.95	5.82

#### 2.2.4 稳定性

取尿液样品加入低(2,3-dinor-TXB_2_,0.20 ng/mL;其余3种化合物,0.90 ng/mL)、高(2,3-dinor-TXB_2_,16 ng/mL;其余3种化合物,8 ng/mL)两水平的混合标准溶液,每个水平4个平行样品,并进行前处理,分别考察样品在进样器(4 ℃)放置24 h和-70 ℃放置10 d的稳定性。结果显示,在上述条件下,目标化合物含量的相对误差均在±15%内,表明样品在上述条件下保持稳定。

### 2.3 实际样品检测

采用上述建立的方法对实际样品进行测定,将得到的目标化合物峰面积与其内标比值代入标准曲线得到浓度,并用肌酐校正。用TXA_2_的3种代谢物加和评估体内TXA_2_的含量,结果进行二元Logistic回归分析并绘制ROC曲线(见图2)。8-iso PGF_2α_曲线下面积(AUC)为0.775,曲线上最佳诊断点的灵敏度和特异度分别为84.6%和76.9%; TXA_2_的AUC为0.849,曲线上最佳诊断点的灵敏度和特异度分别为69.2%和92.3%,提示该两类化合物具有缺血性脑卒中筛查能力。

**图 2:**
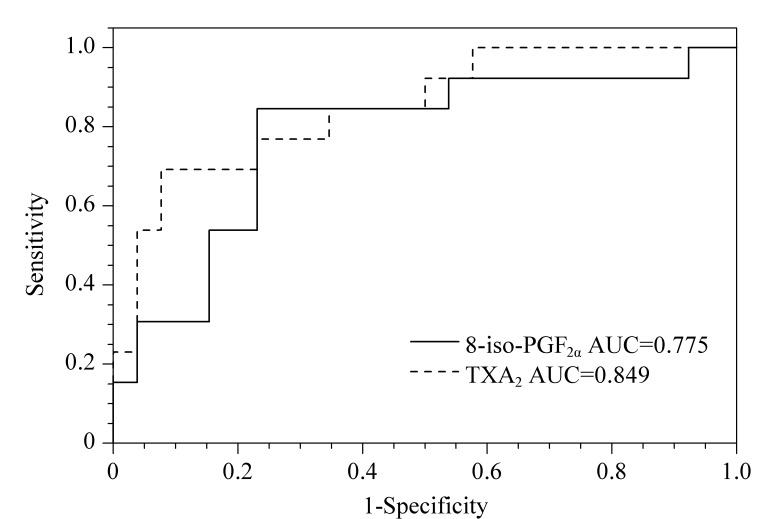
8-iso-PGF_2α_和TXA_2_的ROC曲线

## 3 结论

本文基于超高效液相色谱-串联质谱技术建立了一种同时测定血栓素A_2_的3种代谢物和8-异前列腺素F_2α_的分析方法。该法对色谱及质谱条件进行了优化,评估了方法学指标、基质效应和稳定性,可以实现尿液中目标化合物的准确定量,并在缺血性脑卒中患者中得到验证,有望辅助临床缺血性脑卒中以及其他心血管疾病的早期筛查诊断。
